# Clinical Significance of Blood Cell-Derived Inflammation Markers in Assessing Potential Early and Late Postoperative Complications in Patients with Colorectal Cancer: A Systematic Review

**DOI:** 10.3390/jcm14072529

**Published:** 2025-04-07

**Authors:** Irina Shevchenko, Dragos Serban, Laurentiu Simion, Ion Motofei, Bogdan Mihai Cristea, Dan Dumitrescu, Corneliu Tudor, Ana Maria Dascalu, Crenguta Serboiu, Laura Carina Tribus, Andrei Marin, Adrian Marius Silaghi, Daniel Ovidiu Costea

**Affiliations:** 1 Faculty of Medicine, Carol Davila University of Medicine and Pharmacy Bucharest, 020021 Bucharest, Romania; irina.shevchenko@drd.umfcd.ro (I.S.); laurentiu.simion@umfcd.ro (L.S.); dan.dumitrescu@umfcd.ro (D.D.);; 2 Fourth Department of General Surgery, Emergency University Hospital Bucharest, 050098 Bucharest, Romania; 3 Department of Surgical Oncology, Institute of Oncology “Prof. Dr. Al. Trestioreanu”, 022328 Bucharest, Romania; 4 Department of Surgery, “Sf. Pantelimon” Emergency Hospital, 021659 Bucharest, Romania; 5 Faculty of Dental Medicine, Carol Davila University of Medicine and Pharmacy Bucharest, 020021 Bucharest, Romania; 6 Department of Gastroenterology, Ilfov Clinic Hospital, 022104 Bucharest, Romania; 7 Plastic Surgery Department, “Sf. Ioan” Emergency Clinical Hospital, 042122 Bucharest, Romania; 8 Faculty of Medicine, ‘Ovidius’ University, 900470 Constanta, Romania; 9 Department of General Surgery, Emergency County Clinic Hospital, 900591 Constanta, Romania

**Keywords:** colorectal cancer, postoperative complications, neutrophil-to-lymphocyte ratio, platelet-to-lymphocyte ratio, systemic immune–inflammation index, lymphocyte-to-monocyte ratio

## Abstract

**Background/Objectives**: Colorectal cancer (CRC) is one of the most prevalent malignancies worldwide. Despite advancements in surgical techniques and oncological treatments, postoperative complications remain a significant challenge, affecting both immediate recovery and long-term survival. Systemic inflammation has been identified as a critical factor influencing cancer progression and postoperative outcomes. This systematic review evaluates the clinical significance of blood cell-derived inflammatory markers in predicting early and late postoperative complications in CRC patients. **Methods**: We included studies involving adult patients (≥18 years) with histologically confirmed colorectal cancer, for whom elective radical surgery was performed, as well as at least one of the considered blood-based inflammatory biomarkers (NLR, PLR, SII, or LMR) documented in relation to outcomes. **Results**: After removing duplicates, 19 studies published between 2016 and 2025 were included in the qualitative analysis. A total of 7023 patients who underwent elective curative surgery for colorectal cancer were analyzed, with mean age varying widely between 47.3 and 74.6 years. Preoperative NLR values were significantly correlated with severe complications, IAI, AL, and overall complications in 13 out of 19 studies (68.4%), with a cutoff value between 2.21 and 4, while early postoperative NLR values were predictive for AL. Late postoperative complications, such as recurrence and overall survival, were also associated with elevated preoperative NLR and PLR values. However, variability in study designs, patient populations, and cutoff values for these indices contributed to inconsistent findings. **Conclusions**: Blood cell-derived inflammatory markers offer a valuable, non-invasive tool for assessing postoperative risks in patients with CRC. New design nomograms or risk scores that include, beside blood cell-derived inflammation markers, other relevant data, could ensure an optimal predictive value that could be easily used in clinical practice for personalized risk management in patients with colorectal cancer.

## 1. Introduction

Colorectal cancer (CRC) remains a significant global health concern, ranking third among all diagnosed cancers and second in cancer-related mortality. Among patients under 50 years old, it is the fourth leading cause of cancer-related deaths [1,2,3]. Notably, CRC has shown a concerning trend in younger populations, with a steady 2% annual increase in incidence among individuals under 50 since the 1990s [4,5,6]. In Europe, the projected CRC-related deaths for 2024 were estimated to reach 86,509 cases, with an Age-Standardized Rate (ASR) of 14.74 [1,4]. Given its rising incidence and substantial mortality rates, CRC has been the subject of extensive research, leading to the development of various theories regarding its pathogenesis [7,8,9,10]. These include genetic mutations, tumor histopathology, and the role of the immune system in disease progression [11,12,13]. While the immune system’s involvement in CRC initiation and progression is well documented, the impact of chronic inflammation accompanying neoplasms on postoperative complications remains largely theoretical [14,15,16,17]. This knowledge gap underscores the necessity for an in-depth examination of laboratory data to uncover clinically significant correlations [18,19]. Researchers have approached this challenge in multiple ways; for example, some have focused on detecting specific inflammatory markers directly, while others have relied on calculated inflammation indices using specialized formulas based on theoretical models [20,21,22]. These approaches have led to the emergence of various inflammatory indices, including the Neutrophil-to-Lymphocyte Ratio (NLR), Platelet-to-Lymphocyte Ratio (PLR), and Systemic Immune–Inflammation Index (SII). While these indices have been assessed in clinical settings, their effectiveness in predicting postoperative complications remains unconfirmed [23,24,25,26,27]. This growing body of evidence highlights the need for a systematic literature review to explore the relationship between inflammatory indices and postoperative complications in patients with CRC. Of particular interest, there are associations among the NLR, PLR, and SII; infectious complications; and late postoperative complications.

To address these challenges, we performed a systematic review to evaluate the correlations and the predictive value of blood-based systemic inflammatory markers for postoperative complications after radical surgery in colorectal cancer.

## 2. Materials and Methods

### 2.1. Search Strategy

We conducted a comprehensive literature search across the PubMed, Cochrane Library, Embase, Science Direct, and Springer Nature databases to identify studies examining the association between blood-based systemic inflammatory biomarkers (NLR, PLR, SII, and LMR) and the onset of early and late postoperative complications in patients with colorectal cancer who underwent radical surgery. The search was designed to maximize sensitivity while maintaining specificity through a combination of Boolean operators, database-specific filters, and manual reference screening.

The search strategy was based on the PRISMA review strategy, screening terms related to systemic inflammation, colorectal cancer, and postoperative complications, using AND to combine key concepts and OR to capture synonyms and variations in terminology. The core query included terms such as “inflammatory indices” AND/OR “Neutrophil-to-Lymphocyte Ratio” AND/OR “NLR” AND/OR “Platelet-to-Lymphocyte Ratio” AND/OR “Systemic Immune-Inflammation Index” AND/OR “SII”, in conjunction with “colon cancer” AND/OR “rectal cancer “AND/OR “colorectal cancer” AND/OR “CRC”, as well as outcome-related terms such as “early postoperative complications” AND/OR “surgical complications”.

To refine the selection process, additional filters were applied, limiting the results to randomized controlled trials (RCTs), cohort studies, and observational studies, with only full-text articles considered for inclusion. All English language articles for which full texts could be obtained were screened for eligibility. Although no restrictions were placed on the initial date of publication, the search was concluded in January 2025 to ensure the inclusion of the most recent findings.

**Figure 1:**
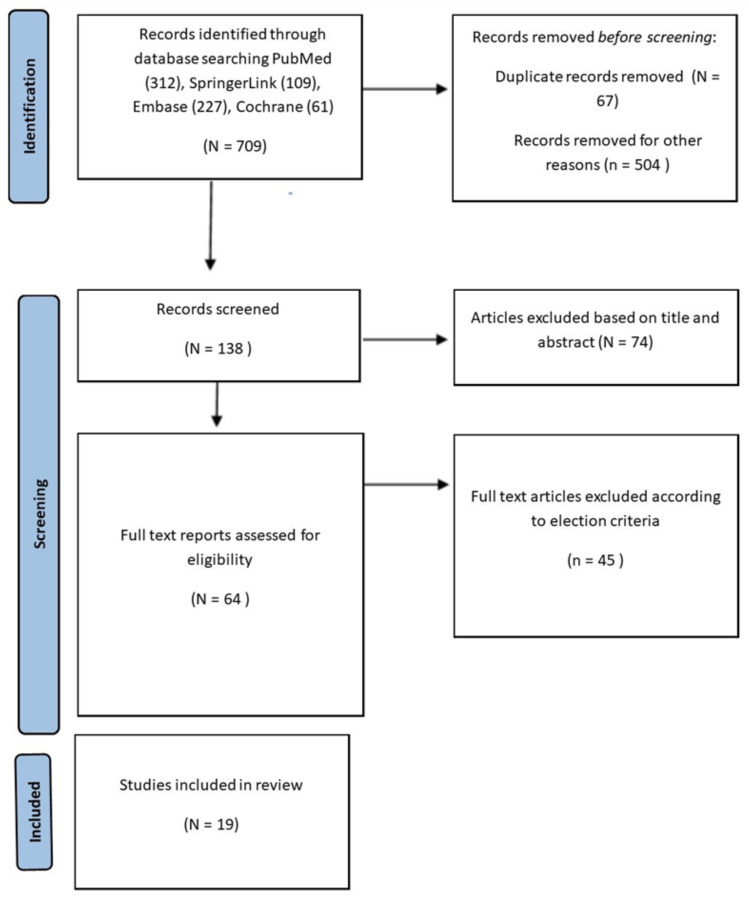
Flow diagram of preferred reporting items for systematic reviews and meta-analyses.

Article selection followed the standardized PRISMA framework [28]. A flowchart of the selection process is presented in Figure 1.

Additionally, the PICO framework was used to structure the systematic search and selection of eligible publications investigating the relationship between inflammatory indices and postoperative complications in patients with colorectal cancer [29,30]. The inclusion criteria consisted of the following: studies involving adult patients (≥18 years) with histologically confirmed colorectal cancer, for whom elective radical surgery was performed, and reports of at least one of the blood-based inflammatory biomarkers (NLR, PLR, SII, LMR) documented in relation to outcomes. Studies involving both pre- and postoperative values for the biomarkers of interest were included. Studies with insufficient documentation or involving metastatic colorectal cancer and palliative surgery were excluded from the analysis. In addition, studies reporting data for patients with colorectal cancer operated on in emergencies were excluded to ensure the consistency of the reported data. 

This approach ensures clarity and precision in identifying relevant studies (Table 1).

### 2.2. Inclusion and Exclusion Processes 

To ensure the selection of high-quality studies with robust statistical power, we applied a structured approach to article inclusion and exclusion. Articles were considered for inclusion if they met the following conditions: full-text availability; clear and detailed statistical methods: studies were required to provide a transparent description of their statistical approaches, including effect size calculations, significance testing, and multivariate adjustments where applicable; well-defined research conditions: the study design, patient recruitment criteria, and inclusion/exclusion conditions had to be explicitly stated to confirm methodological rigor; and a broad range of clinical data: only studies that provided comprehensive patient characteristics, including demographics, tumor staging, treatment strategies, and inflammatory indices, were included to facilitate meaningful comparisons.

Studies were excluded if they met any of the following conditions: case reports or case series: due to their limited generalizability, single-patient case reports and small case series were excluded from the systematic review; insufficient or incomplete methodological descriptions: studies that lacked detailed explanations of data collection, statistical processing, or inclusion/exclusion criteria were deemed ineligible; non-colorectal neoplasms: research focusing on other cancer types, such as gastric, pancreatic, or esophageal cancer, was excluded unless specific colorectal sub-group analyses were provided.

### 2.3. Data Extraction and Quality Assessment of the Obtained Information

Information was systematically extracted from the selected scientific studies following a predefined data collection scheme. This structured approach ensured consistency and accuracy in gathering key details from each publication. The extracted data included the author’s name; year of publication; sample size; tumor location (intestinal/rectal neoplasms); mean age of the study population; gender ratio; cancer stage; type of surgical procedure; presence of neoadjuvant chemotherapy and/or radiotherapy; types of inflammatory markers analyzed; and early and late postoperative complications, including recurrence and mortality rates.

To ensure reliability, data extraction was conducted independently by two researchers. Any discrepancies identified between the extracted data were reviewed and resolved through discussion. In cases where disagreements persisted, a third senior investigator was consulted to reach a consensus. This approach minimized potential bias and enhanced the accuracy of the collected data. Additionally, to verify the integrity of the extracted information, a random subset of studies was re-evaluated to confirm consistency across multiple reviews.

**Figure 2:**
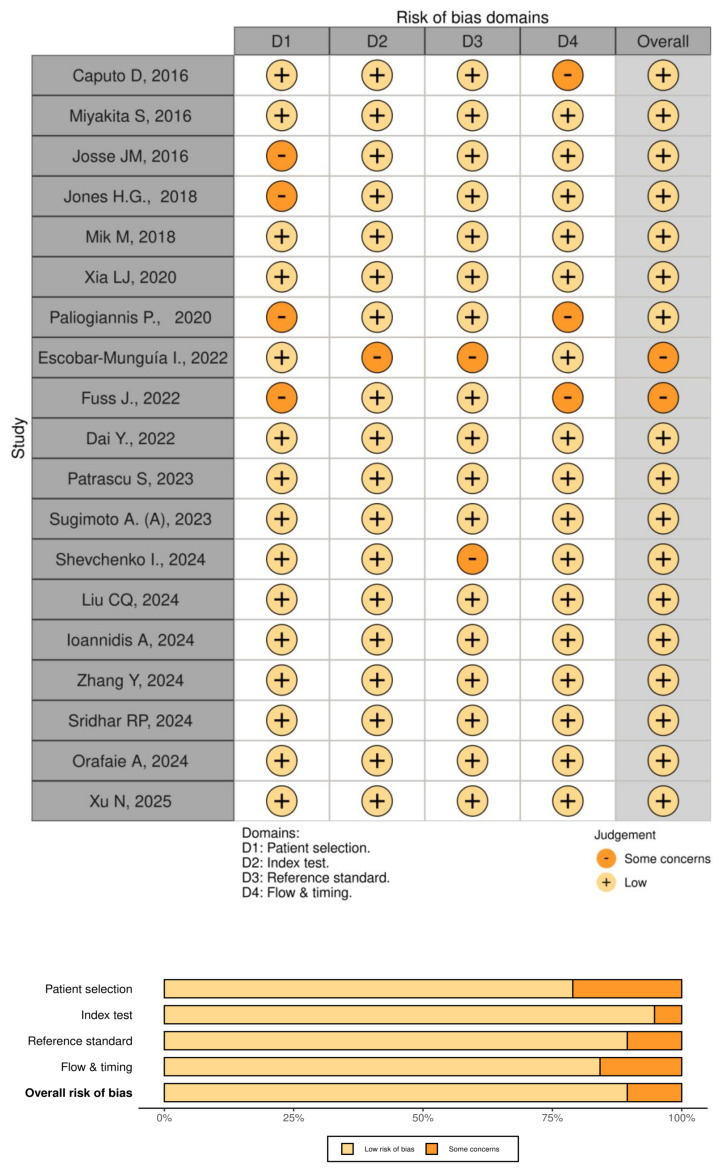
QUADAS-2 (Quality Assessment of Diagnostic Accuracy Studies 2) [35,36,37,38,39,40,41,42,43,44,45,46,47,48,49,50,51,52,53].

For quality assessment, the standardized MINORS score and STROBE system [31,32,33], as well as QUADAS 2 (Quality Assessment of Diagnostic Accuracy Studies 2) (Figure 2) [34] were used to evaluate the accuracy and completeness of the data presented in the selected scientific publications. According to this checklist, the articles were assessed based on 15 criteria, confirming the evidence base’s strength [31,32,33] (Table 2).

Furthermore, QUADAS 2 framework allowed us to systematically evaluate the risk of bias and applicability concerns across four key domains: patient selection, index test, reference standard, and flow and timing. Each study was assessed independently by two reviewers, and any discrepancies in the evaluation were resolved through discussion or consultation with a third senior investigator.

### 2.4. Statistical Analysis

All relevant demographical, clinical, and paraclinical data regarding the patients in the reviewed studies were qualitatively and quantitatively analyzed. The data were analyzed using Microsoft Excel and the Med Calc^®^ Statistical Software (version 22.006, Med Calc Software Ltd., Ostend, Belgium). Numeric variables are expressed as means (±SD) and discrete outcomes as absolute and relative (%) frequencies. The risk of bias (RoB) was assessed using Egger’s test and Begg’s test. The heterogenicity of studies was analyzed using Cochran’s Q and I2 tests for inconsistency. A meta-analysis comparing the predictive value of each biomarker was performed based on the area under the ROC curve and standard error for each biomarker using a random effect model (Med Calc^®^ Statistical Software, version 22.006, Med Calc Software Ltd., Ostend, Belgium). A forest plot was generated to display the relevant findings.

## 3. Results

### 3.1. General Characteristics of the Reviewed Publications

After removing duplicates and applying the inclusion and exclusion criteria, 19 studies [35,36,37,38,39,40,41,42,43,44,45,46,47,48,49,50,51,52,53] published between 2016 and 2025 were included in the qualitative analysis. Most were retrospective (fourteen studies, 78.9%), while in four, the research was prospective [39,42,43,50].

Fifteen studies (78.9%) included patients with both colonic and rectal cancer, while four authors specifically analyzed the correlations between blood-derived inflammatory biomarkers in patients operated on for rectal cancer [38,46,51,52]. Blood samples were taken preoperatively in 11 studies (57.8%) [35,36,40,42,45,46,48,51,52,53]. In eight studies (42.1%), both preoperative and postoperative blood-derived biomarker values were analyzed to determine differences in dynamic changes in patients with uncomplicated and complicated perioperative outcomes [37,38,41,43,44,47,49,50]. Finally, Mik et al. [39] analyzed only samples from the fourth postoperative day (Table 3).

The most frequently analyzed blood-derived biomarkers were NLR (19 studies), PLR (14 studies), and, to a lesser extent, LMR (5 studies) and SII (4 studies), either alone or in combination with other clinical and paraclinical data.

The predictive value of blood-derived biomarkers was analyzed for all types of postoperative complications in 10 studies (52.6%) [53], with specific additional interest in different severe outcomes, such as sepsis [47], AL [36,41,45,49], IAI, and bowel obstruction [36]. In two studies (10.5%), only complications with a Clavien–Dindo value ≥3 were considered [35,37]. Jones [38] and Liu [48] analyzed correlations between IAI and septic complications, while Dai et al. [44] specifically analyzed risk factors for postoperative pulmonary complications in elderly patients receiving elective colorectal surgery.

### 3.2. Clinical Characteristics of the Study Groups

A total of 7023 patients who underwent curative surgery for colorectal cancer were analyzed. The mean age varied widely between 47.3 [51] and 74.6 [49]. The gender distribution revealed an overall slight male predominance (4144 patients, 59.01%). In most cases, male patients predominated in the sample, except for two publications for which women were the majority, albeit with only a slight difference [42,47]. In all cases, cancer diagnosis was confirmed via histopathological examination, and the patients were classified as stages I–III; however, the specific proportions of the individuals with early versus advanced cancer varied between studies. Additionally, the study groups often included clinical cases that had undergone neoadjuvant chemotherapy/radiotherapy according to the tumoral stage protocol [35,36,38,46,47,52]. In six studies [37,39,40,45,48,50], preoperative RCT was used as an exclusion criterion, while in the others, no information was provided [41,42,43,44,49,51,53].

### 3.3. The Significance of NLR in Predicting Postoperative Outcomes

While several authors have demonstrated a clear increase in NLR values with colorectal surgery, this can be explained by the effects of operatory trauma and a possible rise in early postoperative complications. Thus, we separately analyzed the findings regarding the significance of preoperative and postoperative NLR values in the reviewed studies. The comparative results are presented in Table 4 and Table 5.

Preoperative NLR values were significantly correlated with severe complications, IAI, AL, and overall complications in 13 out of 19 studies (68.4%), with a cutoff value varying between 2.3 and 4 [37,38]. Moreover, several studies found that preoperative NLR was an independent risk factor for various adverse outcomes when multivariate analysis was performed [40,42] and recommended including it in personalized risk assessment scores. In the long term, higher NLR values were associated with lower OS and DFS values [38,40,52,53]. These findings may be partially explained by the fact that higher preoperative NLR values are encountered in patients who undergo neoadjuvant RCT [35], as well as patients in advanced disease stages, with tumor sizes greater than 5 cm [40,52,53]. Vascular tumor invasion is also associated with higher preoperative NLR values [42]. Josse et al. [37] found a significant correlation between NLR ≥2.3 and major postoperative complications, with a trend demonstrating higher AL incidence (*p* = 0.053). Moreover, in a multivariate analysis, age, male gender, CCI score, and preoperative NLR were significant predictors of adverse postoperative outcomes [37]. In six studies (31.5%), no correlations were found between preoperative NLR and complications [41,46,47,49,51,52]. Two authors [41,49] showed that postoperative NLR values, specifically from day 4, correlated well with AL and infectious complications.

**Figure 3:**
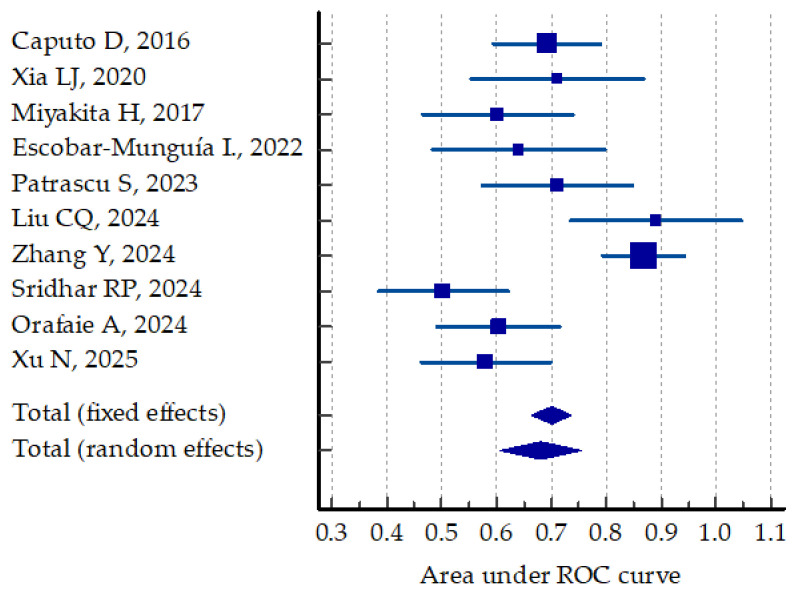
Comparative predictive value of preoperative NLR for postoperative outcomes [35,36,37,40,45,48,50,51,52,53].

The predictive value of preoperative NLR for postoperative complications was analyzed using the ROC curve in 10 studies (55.5%) [35,36,40,42,45,48,50,51,52,53]. The results varied from low to good, with an AUC ROC between 0.581 and 0.890. A comparative meta-analysis based on the AUC and standard error calculated for each study resulted in a random effect AUC ROC of 0.681, as displayed in Figure 3.

**Figure 4:**
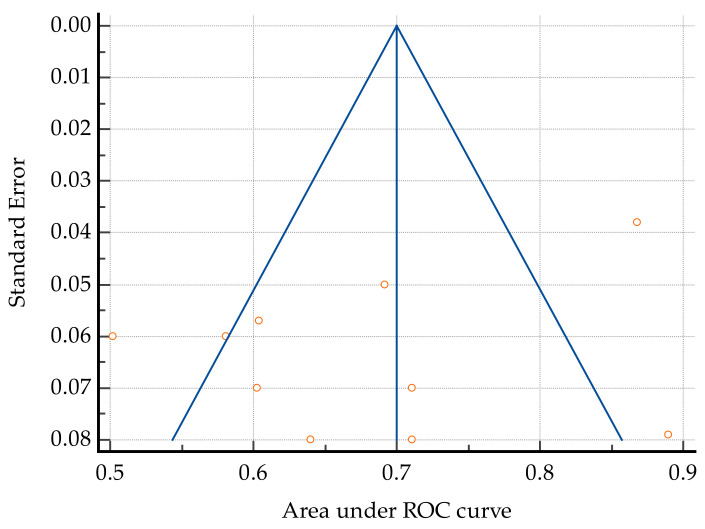
Funnel plot showing increased heterogeneity in the studies included in this review. The oblique blue lines indicate 95% Confidence interval. The vertical blue line depicts the overall effect.

Heterogeneity was high among the studies (Cochrane Q 45.54; I2 = 80.24%, *p* < 0.001); however, the risk of bias was low when applying Egger’s test (*p* = 0.22) and Begg’s test (*p* = 0.85) (Figure 4).

#### The Predictive Value of Postoperative NLR for Postoperative Outcomes

Only seven studies (36.8%) investigated the postoperative NLR values and their potential value in predicting outcomes [37,38,39,41,44,47,49]. The results are presented in Table 5.

Postoperative NLR values were found to be good predictors of postoperative septic complications [37], hospital stay length [37], and the risk of AL [39,41,50]. A valuable insight into the pre- and postoperative dynamic of NLR in complicated versus non-complicated patients was provided by Ioannidis et al. [49], comparing eight daily measurements from preoperative day 1 to day 7 after surgery. The authors found that AL was a good predictor of postoperative NLR values higher than 7.5 at day 1 and higher than 6.5 at day 4. Similar results were reported by Mik et al. [39] and Ioannidis [40]. However, other studies have not found any correlations with the overall risk of complications [37,47].

**Figure 5:**
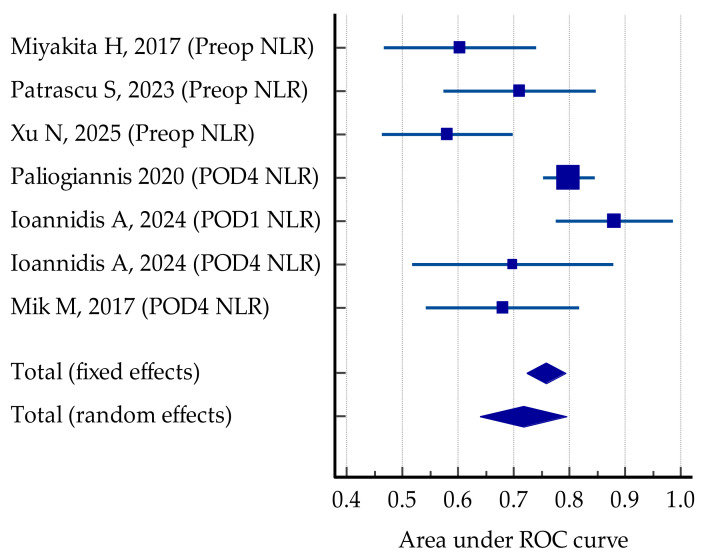
Forrest plot for comparative AUC ROC of pre- and postoperative NLR values in the reviewed studies. Random effect estimation of 0.717 for the risk of AL [36,39,41,45,49,53].

AL is a serious postoperative complication of colorectal surgery, with high morbidity and mortality in oncologic patients. Finding predictive tools to tailor therapeutic management with a personalized approach is a current challenge in colorectal surgery. Six studies specifically analyzed the correlations of pre- and post-NLR values with the incidence of AL [36,39,41,45,49,53]. All authors found good correlations but with variable predictive power; the area under the ROC curve varied between 0.598 and 0.811, with a slightly higher value when postoperative values were considered (Figure 5).

The heterogeneity of the studies was high (I2 = 74.82%), which can be partially explained by the fact that both pre- and postoperative NLR values were tested as predictors; however, the risk of bias was low (*p* = 0.14, Egger’s test; *p* = 0.51, Begg’s test).

### 3.4. The Significance of PLR in Predicting Postoperative Outcomes

A total of 14 studies were included in this analysis to evaluate the predictive value of the pre- and postoperative PLR levels for postoperative outcomes in CRC surgery [35,38,40,41,42,44,45,46,47,48,50,51,52]. Both preoperative and postoperative PLR values were assessed, with results stratified by complication type, severity, and time of onset. Across studies, mean PLR values were consistently higher in groups experiencing complications than in control groups. However, the predictive accuracy and clinical utility varied significantly, with conflicting results, and it was generally considered inferior to that of NLR (Table 6).

Of the reviewed publications, only three authors (25%) did not find PLR useful as a predictive marker for assessing the risk of postoperative complications, as confirmed by statistically non-significant results [35,42,52]. However, Orofaie et al. [52] found that PLR may predict recurrence at a cutoff value of ≥136. Interestingly, similar findings were reported by Xia et al. [40], who found that a preoperative PLR ≥ 140 was correlated with poor OS and DFS at 3 years.

Paliogianis and Xu [41,53] found higher preoperative PLR to be a risk factor for AL. However, Patrascu et al. [45] noted an association between PLR and overall postoperative complications [45] but not with AL. Dai et al. [44], in turn, noted that preoperative PLR reflected the risk of developing pulmonary complications (*p* = 0.014), although the postoperative values did not demonstrate statistical significance (*p* = 0.278).

**Figure 6:**
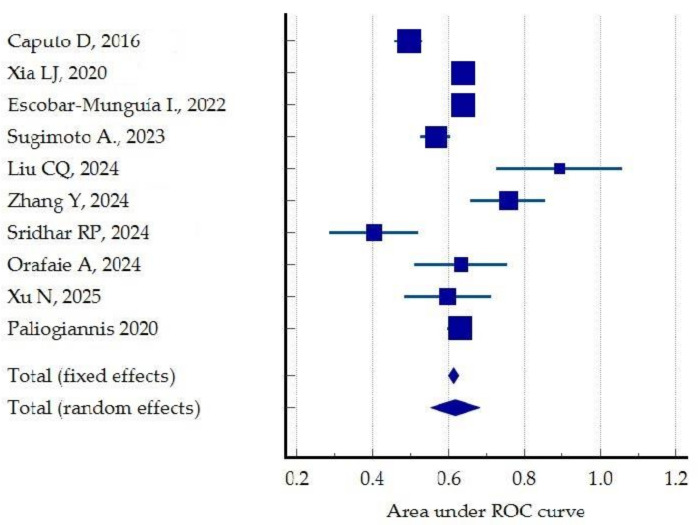
Comparative forest plot for the predictive value of PLR for postoperative outcomes (comparison of AUC ROC) [35,37,40,41,46,48,50,51,52,53].

In the remaining cases, the authors reported favorable outcomes regarding the effectiveness of PLR, although the statistical results varied considerably, with AUC values ranging widely from 0.5 to 0.842 [48,51], albeit slightly lower than those obtained for NLR. A meta-analysis of the comparative outcomes reported in the reviewed studies was performed by comparing the area under the ROC curves. The results of the different studies with a 95% CI and a pooled area under the ROC curve with a 95% CI are shown in a forest plot, with an estimated random effect AUC ROC of 0.618 in the prediction of postoperative complications (Figure 6).

**Figure 7:**
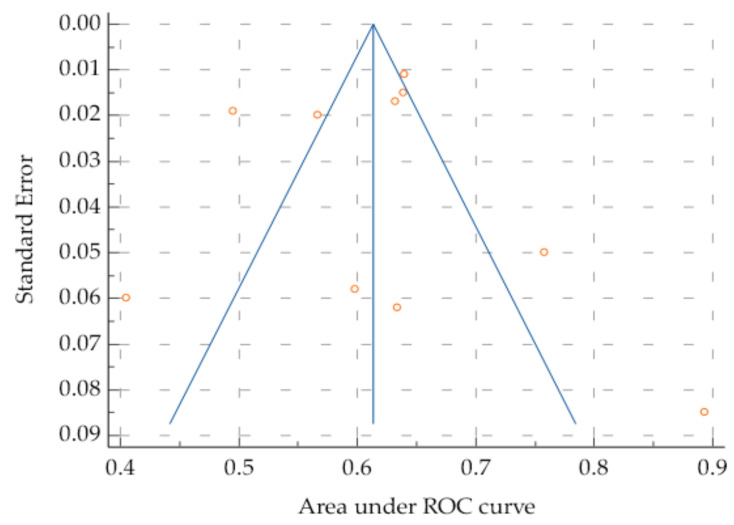
Funnel plot showing increased heterogeneity of the studies included in the review regarding PLR. The oblique blue lines indicate 95% Confidence interval. The vertical blue line depicts the overall effect.

The studies show increased heterogeneity (I2 test: I = 89.49%; Cochrane Q: 85.599), but a reduced risk of publication bias (*p* > 0.05, Egger’s and Begg’s tests). This heterogeneity can be explained in part by the fact that main outcomes vary in the reviewed studies, with some authors considering all complications, while others only cover the major ones (Clavien–Dindo ≥ 3), or even specific subtypes, such as AL, IAI, and infectious or pulmonary complications (Figure 7).

### 3.5. The Predictive Value of SII in the Reviewed Studies

While less investigated than NLR and PLR, preoperative SII seems to be a valuable biomarker for predicting postoperative outcomes, particularly infectious complications. Higher preoperative SII values are correlated with postoperative IAI, with an excellent prediction value (AUC ROC, 0.937) at a cutoff of 826.24 [48]. Similar values are associated with an increased risk of AL and overall complications [45], although Dai et al. [44] found a lower cutoff value of 556.1 to be correlated with postoperative pulmonary complications. Further studies on pre- and postoperative samples could identify the optimal cutoff value for various postoperative outcomes (Table 7).

### 3.6. The Predictive Value of LMR in the Reviewed Studies

LMR was analyzed as a predictive factor for early postoperative outcomes after colorectal radical surgery in four studies [40,45,46,47]. Lower preoperative LMR values were associated with the onset of severe complications—with a Clavien–Dindo value ≥ 3 in two studies [40,46] at a comparable cutoff value (3.9 and 3.48)—and with the risk of reintervention in another study [47]. However, the predictive value was low when evaluated using ROC curves (AUC ROCs of 0.679 and 0.596). However, Patrascu et al. [45] did not find any correlations with AL incidence or overall complications (Table 8).

## 4. Discussions

Colorectal cancer is one of the top causes of cancer-related deaths, despite recent advancements in therapy [54,55,56,57]. Moreover, surgery for colorectal cancer is associated with significant perioperative morbidity, mainly when conducted on an emergency basis [58,59,60]. Recent studies have investigated the preoperative factors that may be correlated with adverse outcomes. A personalized approach, based on comprehensive perioperative evaluation, is essential in reducing the rate of complications and ensuring a safe recovery [61,62].

The significance of the immunological imbalance described by blood cell-derived inflammation markers is still not fully understood in colorectal cancer. Multiple points of evidence indicate the role of inflammation and tissue damage, mediated by neutrophils in cancerogenesis. Higher neutrophils are linked with a pro-inflammatory state, tumor growth, immune evasion, angiogenesis, and increased risk of hepatic metastasis in CRC [63,64]. Recent research by Xiong et al. [65] found that neutrophils play an important role in each step of tumor development, premetastatic niche formation, tumor cell migration, intravasation, and extravasation. Due to their increased plasticity, neutrophils may adapt to different cancer microenvironments and may be reprogrammed into a cancer-promoting state, while monocytes recruit regulatory T cells and reduce CD8 + T cell infiltration, further suppressing anti-tumor immunity [64,65]. On the other hand, the lymphocytic response in colorectal cancer is considered a measure of host response to tumoral invasion, and higher levels of several specific subtypes of infiltrating lymphocytes, such as CD57+, CD8+, CD45RO+, or FOXP3+ cells were found to be an independent factor for improved overall survival [66,67]. Several studies found that platelets may favor tumor development, either by expressing pro-angiogenic factors, such as vascular endothelial growth factor (VEGF) and platelet-derived growth factor (PDGF) or by protecting tumoral cells by inhibiting natural killer cells [68].

This systematic review found that preoperative NLR, PLR, and SII may be valuable predictive factors of early and delayed complications in elective surgery for colorectal cancer. Higher preoperative NLR and SII values were correlated with postoperative severe complications, particularly involving infections, such as AL, and IAI. However, there were differences in the cutoff values found by different authors, ranging from 2.21 [36] to 4 [38]. Age, gender, smoking, and associated comorbidities of the patients included in the study groups may explain these findings. Moreover, several studies correlated higher preoperative NLR values with tumoral stage, and neoadjuvant RCT [35,69,70]. Surgical trauma leads to significantly increased values of NLR, with open surgery having a more significant effect compared to the laparoscopic approach [70], with a notable descending trend from day 3 onward [50]. Higher day 4 postoperative values of NLR ≥ 6.5–7 may be a valuable indicator for AL [41,50].

While correlations between preoperative PLR and early outcomes resulted in conflicting results in the reviewed studies, multiple authors reported a higher risk for recurrence and worse OS in patients with preoperative PLR ≥ 140 [40,52]. One explanation may be that higher PLR values are correlated with lower tumor differentiation, deeper tumor depth, and advanced stage. Lu et al. suggested that PLR should be included in the TNM evaluation [68].

Our review has some limitations. Most studies were retrospective, leading to some sources of bias. While all studies included patients with stage I-III CRC, we noticed significant heterogeneity in terms of mean age, comorbidities, and the gender ratio, as well as variations in terms of tumor size and neoadjuvant therapy, which may have influenced the demographic relevance of data. Moreover, there were differences in terms of targeted postoperative outcomes that were taken into account in the statistical analysis.

Future research, on larger groups, is necessary to define the most accurate cutoff value for each specific outcome. Moreover, NLR was found to be an independent risk factor, but its predictive power varied from fair to good in univariate analysis. A new direction of research includes blood cell-derived inflammatory markers in different risk scores to enhance the predictive value: the Naples Predictive Score (GPS), which combines albumin, cholesterol, NLR, and LMR [47,71]; albumin-NLR [72]; systemic inflammation score (SIS), taking into account albumin and LMR [73]; and NLR-PLR combined score [74], which proved to be valuable in clinical trials.

## 5. Conclusions

This systematic review highlighted the clinical significance of inflammatory markers in assessing early and late postoperative complications in patients with CRC. NLR, PLR, and SII have demonstrated potential in predicting postoperative risks, albeit with some variability across studies. While inflammatory markers offer a promising, non-invasive tool for perioperative risk stratification, further research is warranted to optimize their clinical application. New design nomograms or risk scores that include, besides blood cell-derived inflammation markers, other relevant data could ensure an optimal predictive value that could be easily used in clinical practice for a personalized risk management in patients with colorectal cancer.

## Figures and Tables

**Table 1:** PICO strategy for the studies included in the systematic review.

PICO Element	Description	Inclusion Criteria	Exclusion Criteria
Population (P)	Patients with colorectal cancer undergoing surgical treatment	Adults (≥18 years), histologically confirmed colorectal cancer, elective/emergency surgeries	Other cancer types, pediatric patients, concurrent non-cancer-related conditions
Intervention (I)	Assessment of systemic inflammatory markers before and/or after surgery	NLR, PLR, and SII inflammatory indices	Studies not measuring inflammatory indices or lacking relevant biomarkers
Comparison (C)	Patients without significant postoperative complications	Comparisons between those with and without early postoperative complications	Studies lacking clear comparison groups
Outcomes (O)	Postoperative outcomes related to inflammatory status	Early complications (≤30 days), reoperation, mortality, survival	Non-reported complications or insufficiently described outcome measures
Stusy Design	Type of research and reporting quality	RCTs, cohort studies, observational studies, full-text articles, detailed statistical methods	Case reports, sample size < 100, conference abstracts, reviews, editorials

**Table 2:** MINORS (Methodological Index for Non-Randomized Studies) scores for observational studies included in the review.

Study	Clearly Stated Aim	Inclusion of Consecutive Patients	Prospective Data Collection	Endpoints Appropriate to Aim	Unbiased Assessment of Endpoint	Follow-Up Period Adequate	Loss to Follow-Up <5%	Prospective Calculation of Study Size	Total Score (Max 16)
Caputo D, 2016 [35]	2	2	0	2	2	1	2	0	11
Miyakita S., 2016 [36]	2	2	0	2	1	2	2	0	11
Josse JM, 2016 [37]	2	2	0	2	2	2	2	0	12
Jones HG, 2018 [38]	2	2	0	2	2	1	1	0	10
Mik M, 2018 [39]	2	2	2	2	2	2	2	1	15
Xia LJ, 2020 [40]	2	2	0	2	1	1	2	0	10
Paliogiannis P, 2020 [41]	2	2	0	2	2	2	2	0	12
Escobar-Munguía I, 2022 [42]	2	2	2	2	2	2	2	0	14
Fuss J, 2022 [43]	2	2	2	2	2	1	1	0	12
Dai Y, 2022 [44]	2	2	0	2	1	1	2	0	10
Patrascu S, 2023 [45]	2	2	0	2	1	1	2	0	10
Sugimoto A, 2023 [46]	2	2	0	2	2	2	1	0	11
Shevchenko I, 2024 [47]	2	2	0	2	2	2	1	0	11
Liu CQ, 2024 [48]	2	2	0	2	1	1	2	0	10
Ioannidis A, 2024 [49]	2	2	0	2	2	1	1	0	10
Zhang Y, 2024 [50]	2	2	2	2	2	2	2	0	14
Sridhar RP, 2024 [51]	2	2	0	2	1	1	2	0	10
Orafaie A, 2024 [52]	2	2	0	2	2	1	1	0	10
Xu N, 2025 [53]	2	2	0	2	1	1	2	0	10

**Table 3:** General data for the studies included in the systematic review.

Study, Year	No. of Patients	Design	Biomarkers	Blood Samples	Tumor Location and Stage	Previous Treatment	Age (Mean, Years)	Women (%)	Early Postoperative Complications (30 Days) Analyzed	Delays and Other Outcomes
Caputo D, 2016 [35]	87	Retrospective	NLR PLR dNLR	Before and after neoadjuvant RCT	CRC, stages II–III	CRC stage II, neoadjuvant RCT	67 (38–83)	29 (33%)	All CD ≥ 3	TRG downgrading after nRCT
Miyakita S, 2016 [36]	260 (56; 204)	Retrospective	NLR PNI E-PASS CRS CR-POSSUM SAS	Preoperative (pre CHT)	CRC, stages I–III	Preop RCT for stages II-III	70 ± 28	70 (28%)	All; subtypes: infectious/intestinal obstruction. CD ≥ 3a; AL of CD ≥3b	-
Josse JM, 2016 [37]	583	Retrospective	NLR	Pre- and postoperative D1	CRC, stages I–III	None	65 ± 12.5	243 (42%)	Major (CD ≥ 3)	-
Jones H.G., 2018 [38]	314 (69; 245)	Retrospective	NLR PLR	preoperative/ postoperative (the highest value)	RC, stages I–IV	CHT + RT	67 (34–90)—controls 69 (29–88)—septic complication group	113 (35,9%)	Subtype: septic	OS at 80 days
Mik M, 2018 [39]	724 (33; 691)	Prospective	NLR CRP	Postoperative, day 4	CRC, stages I–III	None	62.4 ± 12	361(49.8%)	AL; death (CD = 5)	-
Xia LJ, 2020 [40]	154	Retrospective	NLR PLR LMR PNI	Preoperative	CRC, stages I–II	None	63.7 (32–90)	64 (41.6%)	All	3-year OS and DFS
Paliogiannis P., 2020 [41]	1432 (106; 1326)	Retrospective	NLR dNLR LMR PLR	Preoperative; postoperative (days 1 and 4)	CRC, stages I–III	NA	65.8 ± 13.7	615 (42.95%)	AL	-
Escobar-Munguía I., 2022 [42]	158 (77; 81)	Prospective	NLR PLR	Preoperative	CRC, stages II–IVA	NA	60.6 ± 13.6 (complications) 59.9 ± 12.5 (control)	83 (52,5%)	All	-
Fuss J., 2022 [43]	234 (59; 175)	Prospective	NLR PLR	Preoperative	CRC, stages I–IV	NA	72.8	109 (46.6%)	Septic complications	-
Dai Y., 2022 [44]	638 (38; 600)	Retrospective	NLR PLR RDW SII	Preoperative; postoperative	CRC, no info	NA	68.5 ± 6.0	220 (34.5%)	Pulmonary complications within 7 days	-
Patrascu S, 2023 [45]	281 (24; 257)	Retrospective	NLR PLR SII LMR	Preoperative	CRC, stages I–III	None	68.70 ± 10.55	121 (43%)	All; subtype: AL	-
Sugimoto A., 2023 [46]	235 (64; 171)	Retrospective	NLR LMR PLR CAR GPS PNI CONUT NPS	Preoperative	RC, stages I–III	CHT + RT	69 (62−75)	78 (33.1)	All	-
Shevchenko I., 2024 [47]	200 (67; 133)	Retrospective	NLR PLR MLR SII	Preoperative; postoperative (days 1, 4, and 6)	CRC, stages I–IV	CHT-RT	68 ± 10.1	102 (51%)	All; subtype: sepsis	-
Liu CQ, 2024 [48]	80 (15; 65)	Retrospective	NLR PLR SII CEA	Preoperative	CRC, stages I–III	None	59.2 ± 12.9	34 (42.5%)	IAI at 30 days	-
Ioannidis A, 2024 [49]	245 (28; 217)	Retrospective	NLR	Preoperative; postoperative (day 0 to day 7–8 measurements)	CRC, stages I–III	NA	74.6 ± 12.0 (L group) 74.7 ± 12.4 (non-L group)	94 (38.3%)	AL	AL at 3 months
Zhang Y, 2024 [50]	109 (31; 78)	Prospective	NLR PLR CRP	Pre- and postoperative (day 2)	CRC, stages I–III	None	62.52 ± 5.68 (complicated) vs. 61.94 ± 6.32 (non-complicated)	44 (40.3%)	Significant, including AL, SSI, intestinal obstruction, abdominal distension, diarrhea, pulmonary infection, urinary tract infection, intra-abdominal infection, and atelectasis	-
Sridhar RP, 2024 [51]	199 (99; 100)	Retrospective	NLR PLR PNI	Preoperative	RC, no info	NA	47.3 years	57 (28.6%)	All	-
Orafaie A, 2024 [52]	200	Retrospective	NLR PLR	Preoperative	RC, grades I–III	Preoperative RCT in all cases	54.2 ± 13.8	84(42%)	All	3-year OS and DFS
Xu N, 2025 [53]	890 (102; 788)	Retrospective	NLR PLR	Preoperative	CRC, stages I–IV	NA	68.66 ± 12.66	358 (40.2%)	AL	OS at 5 yrs

Footnote: AL: anastomotic leak; IAI: intra-abdominal infections; OS: overall survival; DFS: disease-free survival; CRC: colorectal cancer; RC: rectal cancer; nRCT: neoadjuvant radio-chemotherapy; TRG: tumor regression grading; PNI: Prognostic Nutritional Index; CONUT: Controlling Nutritional Status Index; E-PASS CRS: Estimation of Physiologic Ability and Surgical Stress Comprehensive Risk Score; CR-POSSUM: Colorectal Physiological and Operative Severity Score for the Enumeration of Mortality and Morbidity. SAS: Surgical Apgar Score; GPS: Glasgow Prognostic Score; NPS: Naples Prognostic Score; CAR: CRP-to-albumin ratio; NA: no info.

**Table 4:** The value of preoperative NLR in predicting postoperative outcomes after colorectal oncological elective surgery.

Author, Year	Mean Pre-Op NLR in Complications Group	Mean Pre-Op NLR in Controls	Odds Ratio	Pre-Op NLR “Cutoff” Value	AUC ROC	Sensitivity	Specificity	PPV	NPV	Main Findings
Caputo D, 2016 [35]	NA	NA	NA	All postoperative complications: ≥2.8 (pre-nCHT) ≥3.8 (post-nCHT)	Pre-nRCT: 0.476 (*p* = 072) Post-nRCT: 0.693(*p* = 0.006)	Pre-nRCT: NS Post-nRCT: 75%	Pre-nRCT: NS Post-nRCT: 80%	NA	NA	Pre-nRCT: NS Post-nRCT ≥ 3.8 correlated with all complications (*p* = 0.3) and complications with CD ≥ 3 (*p* = 0.049); TRG ≥ 4 (*p* = 0.033).
Miyakita S, 2016 [36]	NA	NA	2.5 (all complications) 3.65 (infectious complications) 4.51 (AL)	≥2.21 (for AL)	NA	83.3% (for AL)	47.4% (for AL)	16.1% (for AL)	95.9% (for AL)	Pre-NLR: Significantly related to incidences of all complications (*p* = 0.003), infectious complications (*p* = 0.013), and AL (0.032) Pre-NLR: The only preoperative independent risk factor for AL.
Josse JM, 2016 [37]	NA	NA	Major complications CD ≥ 3: 2.52 (*p* = 0.02) AL: 2.96 (*p* = 0.053, NS)	≥2.3	NA	NA	NA	NA	NA	Preop NLR ≥ 2.3 increases risk for major complications CD ≥ 3 (*p* = 0.02), but not to a specific subtype.
Jones HG, 2018 [38]	4.82 ± 0.43	3.35 ± 0.11	2.38 (for septic complications, *p* = 0.003) 1.96 (for OS, *p*= 0.006)	≥4 (for septic complications)	NA	33.33% (for septic complications)	83.70% (for septic complications)	46.38% (for septic complications)	74.80% (for septic complications)	Preop NLR ≥ 4 predicts postop septic complications and poor OS.
Xia LJ, 2020 [40]	NA	NA	NA	≥2.8 (all complications, 3 yr OS, DFS)	0.711 (3 yr OS)	53% (3 yr OS)	71% (3 yr OS)	NA	NA	Preop NLR ≥ 2.8 is an independent risk factor for complications (*p* < 0.001) and lower 3 yr OS and DFS (*p* < 0.001).
Paliogiannis P., 2020 [41]	3.30 (2.28–4.13)	2.90 (2.10–3.90)	NS	NS	NS	NA	NA	NA	NA	Preop NLR does not predict AL.
Escobar-Munguía I., 2022 [42]	NA	NA	2.24 (for all complications	≥2.6	0.66 (for all complications)	66.2% (for all complications)	50.6% (for all complications)	NA	NA	Preop NLR ≥ 2.6 increases risk of postoperative complications (*p* = 0.016) and is an independent risk factor.
Fuss J., 2022 [43]	NA	NA	9.827 (for septic complications)	≥3 (for septic complications)	NA	NA	NA	NA	NA	Preop NLR ≥ 3 is an independent risk factor for septic complications (*p* = 0.016).
Dai Y., 2022 [44]	2.5 (1.7, 3.7)	2.1 (1.6, 2.8)	1.193	NA	NA	NA	NA	NA	NA	Higher preop NLR is associated with a risk of postop pulmonary complications, but its value is lower than that of preop SII.
Patrascu S, 2023 [45]	6.73 ± 5.54 (AL) 7.57 ± 4.62 (CD 1,2) 7.66 ± 6.55 (CD 3,4) 6.69 ± 6.90 (CD 5)	3.17 ± 1.7 (NAL) 3.15 ± 1.58 (no complications)	3.159 (AL) 1.104 (overall complications)	≥2.998 (AL) ≥ 3.26 (overall complications)	0.711 (AL) 0.774 (overall complications)	68.2% (AL) 73.6% (overall complications)	56.1% (AL) 62.4% (overall complications)	NA	NA	Higher preop NLR correlates with a higher incidence of AL (*p* = 0.009) and overall complications (0.001).
Sugimoto A., 2023 [46]	2.22 (1.73−3.20)	2.12 (1.60−2.99)	NA	≥2.81	0.542	40.6%	71.4%	NA	NA	Preop NLR is not significantly associated with overall complications (*p* = 0.155).
Shevchenko I., 2024 [47]	NA	NA	NS	NA	NA	NA	NA	NA	NA	Preop NLR does not correlate with severe complications.
Liu CQ, 2024 [48]	3.43 ± 0.65	2.08 ± 0.84	1.199	≥2.67	0.890	86.7%	80%	NA	NA	Preop NLR ≥ 2.67 is a good predictor of IAI (*p* = 0.001).
Ioannidis A, 2024 [49]	4.81 (2.96)	4.88 (4.72)	NS	NA	NA	NA	NA	NA	NA	Preop NLR is not correlated with AL (*p* = 0.19).
Zhang Y, 2024 [50]	3.41 ± 0.89	2.12 ± 0.75	7.448	≥2.485	0.868	90.3%	66.7%	NA	NA	Preop NLR ≥ 2.485 has a predictive value for postoperative complications.
Sridhar RP, 2024 [51]	4.7 ± 3.8	3.8 ± 2.7	NA	≥2.8	<0.500 (NS)	NA	NA	NA	NA	Preop NLR is not associated with complications (*p* = 0.056).
Orafaie A, 2024 [52]	NA	NA	NA	≥2.69 (mortality) NS for surgical infections	0.604 (mortality)	80.5% (mortality)	42.8% (mortality)	NA	NA	Preop NLR ≥ 2.69 correlates with mortality (at 24-month follow-up) but not with surgical infectious complications.
Xu N, 2025 [53]	3.65 ± 3.19	2.98 ± 2.83	1.790 (AL) 1.676 (OS)	≥ 2.29 (AL) ≥2.61 (OS and DFS)	0.581 (AL) 0.582 (OS and DFS)	63% (AL) 50% (OS and DFS)	55% (AL) 66% (OS and DFS)	NA	NA	Higher preop NLRs are correlated with the risk of AL (0.037) and poor OS and DFS, but the predictive value is low.

Footnote: nRCT: neo-adjuvant radio-chemotherapy; NS: not significant; CD: Clavien Dindo classification of the postoperative complications; AL: anastomotic leaks; NA: not acknowledged; OS: overall survival; DFS: disease free survival.

**Table 5:** Postoperative NLR significance in the reviewed studies.

Author, Year	Mean NLR in Complications Group	Mean NLR in Controls	Odds Ratio	Cutoff Value	AUC	Sensitivity	Specificity	PPV	NPV	Main Findings
Josse JM, 2016 [37]	NA	NA	NS	POD1 NLR ≥3.9	NS	NA	NA	NA	NA	POD1 NLR is not associated with a risk of complications.
Jones HG, 2018 [38]	POD1 6.89 ± 1.64	POD1 12.92 ± 0.57	NA	NA	NA	NA	NA	NA	NA	Higher PO NLR is associated with septic complications (*p* = 0.025) and better predictive values than pre-PLR.
Mik M, 2018 [39]	9.03 ± 4.13 (AL) 10.71 ± 2.08 (death)	4.45 ± 2.25 (no AL) 8.65 ± 4.67 (survived)	NA	6.5	NA	69%	78%	49%	88%	Higher postop NLR values are predictors of AL and mortality (*p* = 0.001).
Paliogiannis P., 2020 [41]	POD1: 9.80 (7.12–12.30) POD4: 9.60 (6.55–10.98)	POD1: 8.35 (6.00–11.80) POD4: 5.30 (3.60–7.40)		≥7.1	0.744 (POD4 NLR for AL)	72.73% (POD4 NLR for AL)	73.44% (POD4 NLR for AL)	NA	NA	Higher postop NLR correlates with AL; POD4 NLR ≥ 7.1 predicts higher risk of AL (*p* < 0.001) better than pre-NLR, POD1 NLR, and PLR.
Dai Y., 2022 [44]	10.1 (7.4,15.9)	8.9 (6.1,13.4)	NS	NA	NA	NA	NA	NA	NA	Postop NLR is not associated with pulmonary complications.
Shevchenko I., 2024 [47]	NA	NA	NS	NA	NA	NA	NA	NA	NA	POD1 and POD4 NLR correlate with length of stay (*p* < 0.001). No correlations with severe complications.
Ioannidis A, 2024 [49]	POD1: 9.37 (2.98) POD4: 8.08 (6.02)	POD1: 5.50 (1.54) POD4: 10.89 (5.16)	NA	≥7.4 (POD1 for AL) ≥6.5 (POD4 for AL)	0.881 (POD1 for AL) 0.698 (POD4 for AL)	68.7% (POD1 for AL) 82.1% (POD4 for AL)	96.4% (POD1 for AL) 51.6% (POD4 for AL)	28.4% (POD1 for AL) 17.6 (POD4 for AL)	99.3 (POD1 for AL) 96.5 (POD4 for AL)	Higher POD1 and POD4–7 values for NLR are predictive of AL (*p* = 0.001).

Footnote: NS: not significant; NA: not acknowledged; POD1: postoperative day 1; POD4: postoperative day 4; PO: postoperative; AL: anastomotic leak.

**Table 6:** The value of PLR in predicting postoperative outcomes.

Author, Year	Mean PLR in Complications Group	Mean PLR in Controls	Odds Ratio	Cutoff Value	AUC	Sensitivity	Specificity	Main Findings
Caputo D, 2016 [35]	Before nRCT: 149 (63–382) After nRCT: 246 (81–1430)		NS	Pre-nRCT ≥ 143 Post-nRCT ≥ 189	Pre-nRCT: 0.405 Post-nRCT: 0.495	NS	NS	Preop PLR is not associated with complications.
Jones J., 2018 [38]	Preop: 284.86 ± 30.4 PO: 296.63 ± 11.82	Preop: 193.00± 6.00 PO: 398.80 ± 39.89	NA	NA	NA	NA	NA	Higher pre- and post-PLR values are associated with postoperative septic complications (*p* = 0.004; *p* = 0.016).
Xia LJ, 2020 [40]	NA	NA	NA	≥140 (for 3 yr OS and DFS)	0.639 (for 3 yr OS and DFS)	80% (for 3 yr OS and DFS)	58% (for 3 yr OS and DFS)	Preop PLR ≥ 140 correlates with higher incidence of complications (*p* = 0.025) and lower 3 yr OS and DFS (*p* = 0.012).
Paliogiannis P., 2020 [41]	Preop: 200 (141–276) POD1: 270 (190–374) POD4: 254 (212–338)	Preop: 178 (129–253) POD1: 230 (158–317) POD4: 218 (154–288)	NA	≥217 (POD4 for AL)	0.632 (POD4 PLR for AL)	74.49% (POD4 PLR for AL)	49.87 (POD4 PLR for AL)	Higher pre- and postop PLR values correlate with the risk of AL (*p* = 0.038; *p* = 0.0009; *p* < 0.0001), but the predictive value is low under ROC analysis.
Escobar-Munguía I., 2022 [42]	NA	NA	NS	≥216.2 (for all complications)	0.64	63.6%	50.6%	Preop PLR is not associated with postoperative outcomes (*p* = 0.07).
Shevchenko I., 2024 [47]	NA	NA	NA	NA	NA	NA	NA	Higher preop and POD1 PLR are associated with severe postoperative complications (*p* = 0.01; *p* = 0.002) and a higher rate of reintervention (*p* = 0.02; *p* = 0.002).
Liu CQ, 2024 [48]	253.87 ± 40.95	177.65 ± 62.28	1.978	≥213.18	0.842	0.933	0.723	Preop PLR ≥ 213.18 has a good predictive value for IAI (*p* < 0.001).
Zhang Y, 2024 [50]	168.75 ± 36.82	131.06 ± 32.49	1.023	≥142.79	0.758	74.2%	67.9%	Preop PLR ≥ 142.79 is a predictor of postoperative complications (*p* < 0.001).
Sridhar RP, 2024 [51]	256.3 ± 178.9	203.4 ± 98.8	NA	≥140	<0.500	NA	NA	Higher preop PLR values are associated with complications (*p* = 0.011), but the predictive value is not significant.
Orafaie A, 2024 [52]	NA	NA	NA	≥136 (recurrence) NS for surgical infections	0.634 (recurrence)	75% (recurrence)	59.7% (recurrence)	Higher preop PLR is correlated with recurrence but not with surgical infectious complications.
Xu N, 2025 [53]	NA	NA	1.803 (AL) 2.081 (OS) 1.202 (DFS)	≥ 133.24 (AL) ≥204.04 (OS and DFS)	0.598 (AL) 0.553 (OS and DFS)	67% (AL) 28% (OS and DFS)	51% (AL) 83% (OS and DFS)	Higher preop NLR values are correlated with the risk of AL (0.037) and poor OS and DFS, but the predictive value is low.

Footnote: NS: not significant; NA: not acknowledged.

**Table 7:** The predictive value of SII for postoperative outcomes after colorectal cancer surgery.

Author, Year	Mean SII in Complications Group	Mean SII in Controls	Odds Ratio	Cutoff Value	AUC	Sensitivity	Specificity	Main Findings
Liu CQ, 2024 [48]	1226.48 ± 245.55	611.52 ± 285.96	1.010	≥826.24	0.937	0.215	0.785	Preop SII ≥ 826.24 has a good predictive value for IAI.
Dai Y., 2022 [44]	Preop SII: 602.5 (347.4, 932.0) Postop SII: 2023.9 (1457.4, 3522.2)	Preop SII: 420.4 (316.8, 645.0) Preop SII: 1734.1 (1099.4, 2717.7)	1.001	≥556.1 (preop SII for pulmonary complications)	0.629 (preop SII for pulmonary complications)	57.9% (preop SII for pulmonary complications)	67.2% (preop SII for pulmonary complications)	Preop SII is an independent predictor for pulmonary complications (0.007); no correlations with postop SII.
Patrascu S, 2023 [45]	1913.19 ± 2368 (AL) 2331.28 ± 2064.51 (mild) 2224.32 ± 2427.79 (moderate) 1936.72 ± 2224.67 (severe)	993.35 ± 878.94 (no AL) 897.99 ± 571.60 (no complications)	0.998	≥793 (AL) ≥933 (overall complications)	0.622 (AL) 0.702 (overall complications)	63% (AL) 66.7% (overall complications)	53% (AL) 61.3% (overall complications)	Higher preop SII correlated with higher incidence of AL (*p* = 0.001) and overall complications (*p* = 0.001).
Shevchenko I, 2024 [47]	NA	NA	NA	NA	NA	NA	NA	POD1 SII correlates well with severe complications (*p* = 0.01).

Footnote: NA: not acknoledged; AL: anastomotic leak.

**Table 8:** The predictive value of LMR in the reviewed studies.

Author, Year	Mean LMR in Complications Group	Mean LMR in Controls	Odds Ratio	Cutoff Value	AUC	Sensitivity	Specificity	Main Findings
Xia LJ, 2020 [40]	NA	NA	NA	≤3.9 (for 3 yr OS and DFS)	0.679 (for 3 yr OS and DFS)	73%	65%	Preop LMR ≤ 3.9 correlates with CD 3,4 complications (*p* = 0.04), lower OS, and DFS at 3 yrs (*p* = 0.002).
Patrascu S., 2023 [45]	2.50 ± 1.65 (AL) 2.09 ± 1.15 (mild) 2.61 ± 1.59 (moderate) 2.70 ± 1.84 (severe)	4.04 ± 3.48 (no AL) 4.50 ± 9.71 (no complications)	NS	NA	NA	NA	NA	No correlations with AL (*p* = 0.06) or overall complications (*p* = 0.1).
Sugimoto, 2023 [46]	4.67 (3.02 − 5.91)	5.26 (4.04 − 6.69)	NA	≤3.48	0.596	37.5%	82.5%	Preop LMR ≤ 3.48 is associated with severe complications (*p* = 0.023), but the predictive value is low.
Shevchenko I, 2024 [47]	NA	NA	NA	NA	NA	NA	NA	Lower preoperative LMR is associated with reintervention (*p* = 0.02).

Footnote: NA: not acknoledged; AL: anastomotic leak.

## Data Availability

No new data were created.

## Abbreviations

NLR	Neutrophil-to-Lymphocyte Ratio
PLR	Platelet-to-Lymphocyte Ratio
SII	Systemic Immune–Inflammation Index
LMR	Lymphocyte-to-Monocyte Ratio
OS	overall survival
DFS	disease-free survival
AL	anastomotic leaks
IAI	intra-abdominal infection
GPS	Glasgow Prognostic Score
CRP	C-reactive protein
NPS	Naples Prognostic Score,
CAR	C-reactive protein/albumin ratio
PNI	Prognostic Nutritional Index
CONUT	Controlling Nutritional Status Index
